# The impact of weight loss in early pregnancy on the incidence of late gestational diabetes: a retrospective cohort study

**DOI:** 10.3389/fnut.2025.1688268

**Published:** 2025-11-17

**Authors:** Zihua Chen, Jiaoxia Liu, Lin Lin, Jun Shi, Huihui Huang, Ruiyun Chen, Qiuping Liao, Liping Huang, Lianghui Zheng

**Affiliations:** 1 College of Clinical Medicine for Obstetrics & Gynecology and Pediatrics, Fujian Maternity and Child Health Hospital, Fujian Medical University, Fuzhou, Fujian, China; 2 Fujian Medical University, Fuzhou, Fujian, China; 3 Fujian Clinical Research Center for Maternal-Fetal Medicine, Fuzhou, Fujian, China

**Keywords:** weight gain, gestational diabetes mellitus, preeclampsia, small for gestational age, preterm birth

## Abstract

**Objective:**

To study the potential correlation between pregnancy weight gain (WG) and the incidence of gestational diabetes mellitus (GDM).

**Methods:**

Clinical records of women with singleton pregnancies who had a first visit at Fujian Maternity and Child Health Hospital before 14 weeks and delivered after 28 weeks were retrospectively analyzed. Based on the first trimester WG, the participants were grouped as inadequate (iWG-F), adequate (aWG-F), and excessive (eWG-F) WG groups. The outcomes of interest included GDM, gestational hypertension, preeclampsia, small for gestational age (SGA), LGA, low birth weight (LBW), preterm birth, macrosomia, primarily cesarean section (CS), and admission to the neonatal intensive care unit (NICU). Statistical analyses included logistic regression, interaction, and mediation analyses.

**Results:**

A total of 16,824 pregnancies were analyzed. GDM incidences of the iWG-F, aWG-F, and eWG-F groups were 24.53%, 26.62%, and 29.46%, respectively, with a statistically significant difference (*p* < 0.001). Multivariable logistic regression showed that inadequate WG correlated with reduced risk of GDM when adjusted for pre-pregnancy body mass index (PPBMI) of below 18.5 kg/m^2^ [adjusted odds ratio (aOR) = 0.68], and 18.5–23.9 kg/m^2^ (aOR = 0.88). The association of inadequate WG and reduced risk of GDM persisted when adjusted for age <30.5 years (aOR = 0.81), fasting glucose ≥4.9 mmol/L (aOR = 0.74), triglycerides <1.4 mmol/L (aOR = 0.84), and HDL <1.63 mmol/L (aOR = 0.85). WG in the second trimester was associated with GDM (*β* = −0.003, *p* = 0.036) and partially mediated the effect of eWG-F (−3.7% of the total effect). WG before OGTT showed no association with GDM.

**Conclusion:**

First trimester WG is significantly associated with the occurrence of GDM. In contrast, there is only a minimal association between second-trimester and pre-OGTT WG and the risk of GDM. Inadequate first-trimester weight gain reduces GDM risk, especially in younger women, women with normal or low PPBMI, elevated fasting glucose, and low HDL or triglycerides, without increasing abnormal neonatal birth weight. Early pregnancy represents a critical window for GDM prevention. Minimal weight gain during this period may be a feasible and acceptable approach to reducing GDM risk.

## Introduction

Gestational diabetes mellitus (GDM) impacts around 14% of pregnant women worldwide (1, 2). A 2018 systematic review reported that a pooled GDM prevalence in mainland China was as high as 14.8% (3), rising to 21% in some regions by 2021 (4). GDM is associated with numerous short-term adverse maternal and offspring outcomes and long-term health risks, including pregnancy-related hypertension, preterm birth, cesarean delivery, large for gestational age (LGA), etc. (5–10). The diagnosis of GDM is typically established between 24 and 28 weeks, and the screening mostly focuses on late gestational diabetes (11). However, recent research suggests that the metabolic changes underlying GDM, such as insulin resistance (12), impaired insulin secretion, or relative insulin deficiency (13, 14), may be detectable as early as the first trimester (before 14 weeks of gestation). This early metabolic dysfunction indicates that early pregnancy could be a crucial period for reducing the incidence of late GDM and its associated complications. Randomized controlled trials suggest that lifestyle interventions with a mean weight gain (WG) reduction of −0.76 kg (95% CI: −1.55, 0.03) during early to mid-pregnancy can reduce the incidence of GDM (15–17). However, the impact of maternal WG during early and mid-pregnancy on GDM risk remains unclear, which prevents the development of effective early preventive strategies. This study aims to study the connection between maternal WG in the first trimester (WG-F), the second trimester (WG-S), and weight gain (WG) before the oral glucose tolerance test (OGTT) and the incidence of late gestational diabetes, providing evidence to guide interventions for improving maternal and neonatal health outcomes.

## Methods

### Study design

This retrospective cohort study, done at Fujian Maternity and Child Health Hospital, spanned two outpatient and inpatient locations in the Gulou and Jinan Districts of Fuzhou, China. Training personnel used electronic medical records to collect participants’ information from their first visit to 3 days postpartum, including weight, medical, and obstetrical data gathered from personal weight records, antenatal clinic visits, and delivery admissions. Medical data were extracted from the healthcare system in August 2024 for analysis. This study received approval from the Ethics Committee of the hospital (2024KY200) and adhered to the Declaration of Helsinki principles (World Medical Association Declaration of Helsinki). No informed consent was needed, as the study utilized anonymized historical medical records data.

### Study population

**Figure 1:**
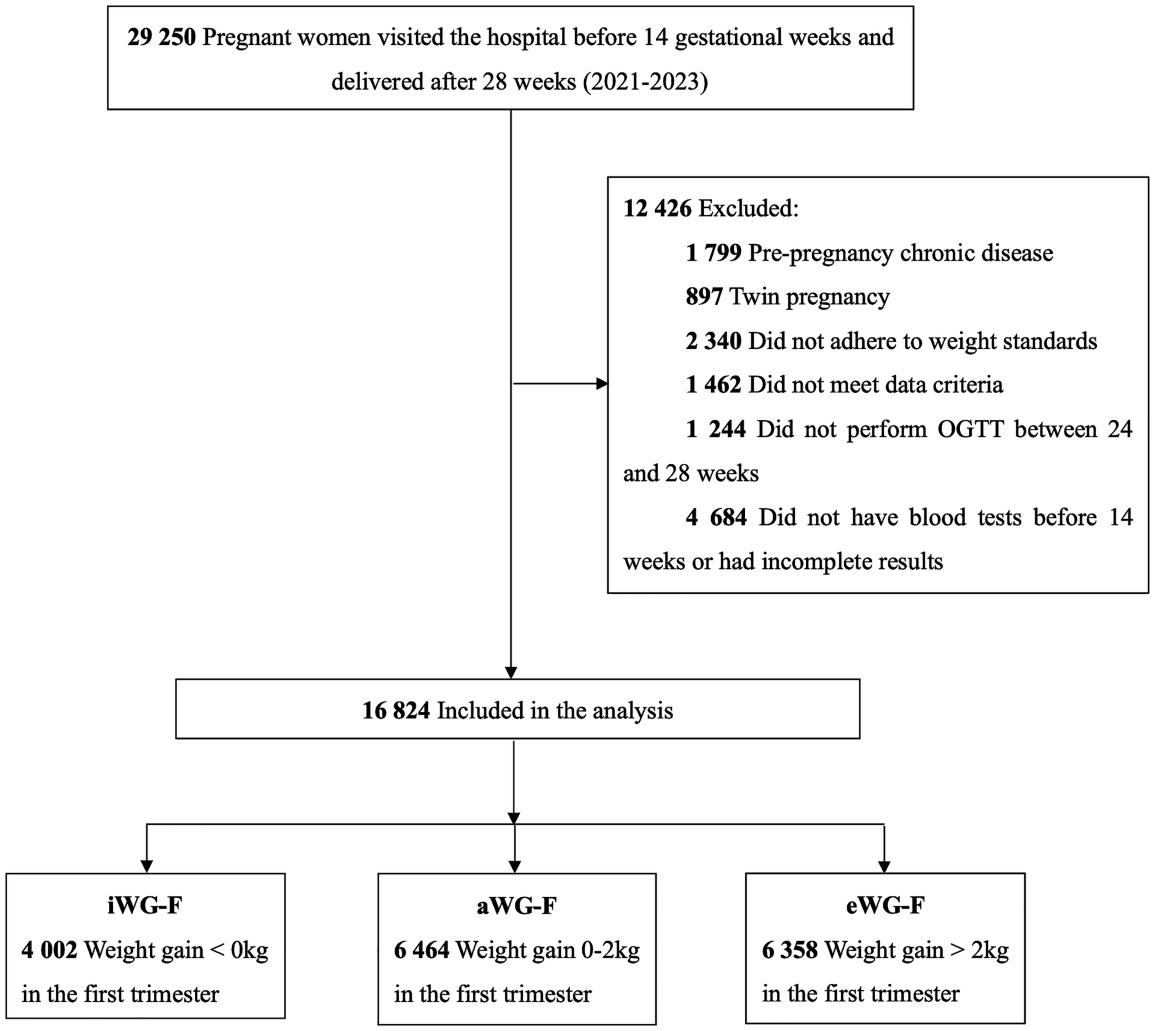
Flow diagram of study participants. iWG-F, inadequate weight gain in the first trimester; aWG-F, adequate weight gain in the first trimester; eWG-F, excessive weight gain in the first trimester.

Patients were enrolled from January 2021 to December 2023. The inclusion criteria were: (1) first visit before 14 gestational weeks, (2) singleton pregnancy, and (3) delivery after 28 weeks. Exclusion criteria were: (1) pre-existing chronic conditions such as heart, kidney, or thyroid disease, type 1 or type 2 diabetes, hypertension, (2) non-compliance with weight monitoring, (3) incomplete documented medical records, (4) OGTT not performed between 24 and 28 weeks, and (5) missing blood test results in the first trimester. According to the weight monitoring and evaluation standards for pregnant women by the Chinese Nutrition Society (18), individuals were categorized into three groups based on WG-F: weight gain <0 kg as inadequate (iWG-F), weight gain between 0 and 2 kg as adequate (aWG-F), and weight gain >2 kg as excessive (eWG-F). (Figure 1).

### Measurements

Weight before pregnancy was self-reported at the initial hospital visit, with concurrent height measurements. Throughout pregnancy, women self-recorded their weight at home on the last day of the 13th week and each subsequent gestational week during the second and third trimesters, following standardized procedures: taken in the morning, after voiding, on an empty stomach, using a precise electronic scale with 0.1 kg accuracy. Trained researchers entered these measurements into the electronic medical record system during subsequent prenatal visits. Pre-delivery weight was the last recorded weight measured at home before delivery or at the hospital at the time of delivery. Gestational weight gain (GWG), WG-F, WG-S, and WG before the OGTT were calculated as follows: GWG, weight just before the delivery - weight before pregnancy; WG-F, weight at the end of 13 weeks - pre-pregnancy weight; WG-S, weight at the end of the week before the OGTT - weight at the end of 13 weeks; and WG before the OGTT, weight at the end of the week before the OGTT - pre-pregnancy weight. The pre-pregnancy body mass index (PPBMI), pre-pregnancy weight (kg)/height (m) squared. For the weight gain, weight gain <0 kg was defined as inadequate (iWG-F), weight gain between 0 and 2 kg was defined as adequate (aWG-F), and weight gain >2 kg was considered excessive (eWG-F).

### Outcomes

The main outcome was GDM. The secondary outcomes included gestational hypertension, preeclampsia, small for gestational age (SGA), large for gestational age (LGA), low birth weight (LBW), preterm birth, macrosomia, primarily cesarean section (CS), and hospitalization in the neonatal intensive care unit (NICU).

### Lifestyle interventions

All pregnant women were guided to adopt healthy dietary and lifestyle habits during gestation. Women received routine prenatal care and dietary guidance from obstetricians and nutritionists at least three times: during the initial visit, mid-pregnancy, and late pregnancy, based on the 2016 Chinese Dietary Guidelines for Pregnant Women (19).

Energy intake was adjusted according to the woman’s pre-pregnancy BMI, physical activity levels, and GWG. In early pregnancy, women without significant morning sickness were advised to maintain a balanced pre-pregnancy diet. At the same time, those with poor appetite or vomiting were recommended to consume a daily minimum of 130 g of carbohydrates, allowing flexibility in food choices. Women with severe hyperemesis gravidarum were advised to seek medical attention. During mid-to-late pregnancy, a daily intake of 300–500 g of dairy products and 150–250 g of fish, poultry, eggs, or lean meat was recommended, with deep-sea fish consumption encouraged 2–3 times per week. Women were advised to take multivitamins containing 0.8 mg of folic acid, 60 mg of iron, and vitamin D as needed, and to engage in moderate-intensity aerobic exercise for 30–40 min, 3–5 times per week, which could be divided into shorter sessions throughout the day.

### Statistical analysis

Studio version 2024.04.2 (Posit Software, PBC) was used for all analyses, including *t*-tests, chi-square tests, cut-off value calculations, logistic regressions, and interaction and mediation. Continuous parameters that follow a normal distribution were expressed as mean ± standard deviation (SD), while non-normally distributed parameters were expressed as medians (range). Forest plots were generated with Prism version 10.2.0 (GraphPad Software, San Diego, CA, USA). Baseline characteristics were compared across the three categories based on the weight gain in the first trimester. Logistic regression was used to analyze the associations between WG categories (iWG-F and eWG-F) and 10 short-term pregnancy outcomes, using aWG-F as the reference group. Covariates included PPBMI, maternal age, delivery weeks, parity, and GWG, except for preterm birth analysis, where delivery weeks were excluded. For GDM analysis, univariate logistic regression calculated odds ratios (ORs) and 95% confidence intervals (CIs) for 13 clinical characteristics. Multivariable logistic regression estimated adjusted odds ratios (aORs) and 95% CIs.

Logistic regression was used to assess the relationships between WG-F, WG-S, and WG before OGTT in patients with GDM. Interaction and mediation analyses explored potential pathways, adjusting for maternal age, PPBMI, parity, education level, gestational age at delivery, fasting glucose, triglycerides, and HDL levels in early pregnancy. Subgroup analyses were performed by stratifying participants by age, PPBMI, fasting glucose, triglycerides, and HDL, with subgroup-specific factors excluded from the confounding variables.

## Results

### Participants’ characteristics

In total, 16,824 pregnancies were enrolled in this study. Of them, 4,002, 6,464, and 6,358 pregnant women demonstrated iWG-F, aWG-F, and eWG-F, respectively, with median weight changes of −1.40, 1.00, and 3.50 kg. Women in the iWG-F group were the youngest, had the highest pre-pregnancy weight and PPBMI, and exhibited the smallest WG-S, WG before OGTT, and total gestational weight gain (all *p* < 0.001). Women in the eWG-F group had the most significant proportion of multiparous women and the lowest educational levels (<9 years) (*p* < 0.001). Early pregnancy blood test results indicated that women in the iWG-F group had the highest fasting glucose and the lowest HDL levels (all *p* < 0.001). No significant differences were observed among the three groups in the prevalence of a history of GDM, type 2 diabetes in first-degree relatives, triglyceride levels, low-density lipoprotein levels in the first trimester, or OGTT results. Detailed baseline characteristics are presented in Table 1.

**Table 1:** Characteristics grouped by weight gain in the first trimester.

Characteristics	Inadequate (iWG-F) *n* = 4 002 (23.79%)	Adequate (aWG-F) *n* = 6 464 (38.42%)	Excessive (eWG-F) *n* = 6 358 (37.79%)	*p* value
Age (years), mean (SD)	29.82 ± 3.84	30.27 ± 4.00	30.54 ± 4.17	<0.001
<35 y at time of birth, *n* (%)	3,562 (89.01)	5,545 (85.78)	5,299 (83.3)	<0.001
≥35 y at time of birth, *n* (%)	440 (10.99)	919 (14.22)	1,059 (16.7)	<0.001
Pre-pregnancy weight (kg), mean (SD)	57.44 ± 9.08	54.87 ± 8.09	54.59 ± 7.72	<0.001
Height (cm), mean (SD)	160.43 ± 5.48	160.15 ± 5.41	160.45 ± 5.41	0.0016
PPBMI (kg/m^2^), P50 (P5, P95)	21.74 (18.03, 28.58)	20.90 (17.47, 26.98)	20.90 (17.31, 26.14)	<0.001
<18.5, *n* (%)	331 (8.27)	937 (14.50)	994 (15.63)	
18.5–23.9, *n* (%)	2,657 (66.39)	4,388 (67.88)	4,445 (69.91)	
24.0–27.9 *n* (%)	772 (19.29)	949 (14.68)	790 (12.43)	
≥28.0, *n* (%)	242 (6.05)	190 (2.94)	129 (2.03)	
Multipara, n (%)	1,426 (35.63)	2,628 (40.66)	2,670 (42.00)	
GDM history, n (%)^a^	10 (0.70)	21 (0.80)	27 (1.01)	0.536
Education year				<0.001
<9 years, *n* (%)	307 (7.67)	408 (6.31)	499 (7.85)	
9–12 years, *n* (%)	394 (9.85)	665 (10.29)	716 (11.26)	
>12 years, *n* (%)	3,301 (82.48)	5,391 (83.40)	5,143 (80.89)	
Type 2 diabetes in first-degree relative, *n* (%)	108 (2.70)	164 (2.54)	145 (2.28)	0.382
Fasting glucose (mmol/L), P_50_ (P_5,_ P_95_)	4.76 (4.53, 5.02)	4.74 (4.51, 4.97)	4.72 (4.50, 4.95)	<0.001
Tiglycerides (mmol/L), P_50_ (P_5,_ P_95_)	1.20 (0.7, 2.18)	1.19 (0.68, 2.19)	1.20 (0.68, 2.23)	0.598
Low-density lipoprotein (mmol/L), P_50_ (P_5,_ P_95_)	2.26 (1.44, 3.4)	2.27 (1.41, 3.38)	2.29 (1.43, 3.38)	0.130
High-density lipoprotein (mmol/L), P_50_ (P_5,_ P_95_)	1.57 (1.12, 2.13)	1.63 (1.17, 2.17)	1.65 (1.18, 2.21)	<0.001
OGTT0 (mmol/L), P_50_ (P_5,_ P_95_)	4.43 (3.92, 5.11)	4.42 (3.92, 5.14)	4.43 (3.92, 5.13)	0.715
OGTT1 (mmol/L), P_50_ (P_5,_ P_95_)	8.02 (5.47, 11.0)	8.08 (5.46, 10.9)	8.08 (5.43, 11)	0.483
OGTT2 (mmol/L), P_50_ (P_5,_ P_95_)	6.78 (4.89, 9.58)	6.76 (4.83, 9.56)	6.77 (4.83, 9.55)	0.952
Gestational weight gain, P_50_ (P_5,_ P_95_)	10.10 (3.40, 17.09)	12.50 (6.60, 19.00)	15.40 (9.20, 23.00)	0.000
Inadequate	632 (15.79)	267 (4.13)	51 (0.80)	
Adequate	2,566 (64.12)	3,800 (58.79)	2,128 (33.47)	
Excessive	804 (20.08)	2,397 (37.08)	4,179 (65.73)	
WG-F, P_50_ (P_5,_ P_95_)	−1.40 (−5, −0.200)	1.00 (0.00, 2.00)	3.5 (2.20, 7.2)	0.000
WG-S, P_50_ (P_5,_ P_95_)	5.80 (0.00, 10.20)	5.80 (2.00, 9.70)	6.00 (2.30, 10.00.4.00)	<0.001
Inadequate	638 (15.94)	690 (10.67)	624 (9.81)	
Adequate	1,218 (30.43)	2,254 (34.87)	2006 (31.6)	
Excessive	2,146 (53.62)	3,520 (54.46)	3,728 (58.6)	
WG before OGTT, P_50_ (P_5,_ P_95_)	4.10 (−3.00, 8.60)	6.90 (2.90, 11.00)	9.85 (5.80, 15.91)	0.000
Inadequate	1,098 (27.44)	757 (11.71)	164 (2.58)	
Adequate	2,455 (61.34)	3,259 (50.42)	1,062 (16.70)	
Excessive	449 (11.22)	2,448 (37.87)	5,132 (80.71)	
Gestational age at delivery, P_50_ (P_5,_ P_95_)	39.00 (37.00, 41.00)	39.00 (37.00, 40.57)	39.00 (36.00, 40.57)	<0.001
Birth weight (g), P_50_ (P_5,_ P_95_)	3220.00 (2540.00, 3895.00)	3240.00 (2570.00, 3875.00)	3285.00 (2580.00, 3930.00)	<0.001

PPBMI, pre-pregnancy body mass index; GDM, gestational diabetes mellitus; OGTT, oral glucose tolerance test; WG, weight gain; WG-F, weight gain in the first trimester; WG-S, weight gain in the second trimester. iWG-F, inadequate weight gain (<0 kg); aWG-F, adequate weight gain (between 0 and 2 kg); eWG-F, excessive weight gain (>2 kg) For continuous parameters, those that follow a normal distribution were expressed as mean (SD), while non-normally distributed parameters were expressed as median (range).

a GDM history ratio is calculated by dividing the number of women with a history of GDM by the number of multiparous. In these three groups, the number of multiparous are 1,426, 2,628, and 2,670, respectively.

### First trimester WG and pregnancy outcomes

Among the three groups, iWG-F had the lowest incidence of GDM (24.53%), primary CS (28.39%), LGA (9.40%), and NICU admission (23.26%). In contrast, this group reported the highest incidence of SGA (7.32%). In contrast, eWG-F had the highest incidence of GDM (29.5%), preterm birth (5.79%), LGA (11.3%), and NICU admission (26.30%) but the lowest incidence of SGA (5.50%) (all *p* < 0.05). After adjusting for PPBMI, maternal age, delivery weeks (excluding preterm birth), parity, and GWG, iWG-F was linked to a lower incidence of GDM (aOR = 0.76, 95% CI 0.69, 0.84), and preterm birth (aOR = 0.76, 95% CI 0.62, 0.92). In contrast, eWG-F was associated with a high incidence of GDM (aOR = 1.37, 95% CI 1.26–1.49) and preterm birth (aOR = 1.66, 95% CI 1.41–1.95). WG-F had no significant association with gestational hypertension, preeclampsia, primary CS, NICU admission, or newborn weight (Table 2).

**Table 2:** The relationship between weight gain in the first trimester and pregnancy outcomes.

Pregnancy outcomes	aWG-F *N* = 6 464	iWG-F *N* = 4 002			eWG-F *N* = 6 358		
	*n* (%)	*n* (%)	aOR (95% CI)^a^	*p*	*n* (%)	aOR (95% CI)^a^	*p*
Gestational diabetes^b^	1721 (26.62)	982 (24.53)	**0.76 (0.69, 0.84)**	**< 0.001**	1873 (29.46)	**1.37 (1.26, 1.49)**	**< 0.001**
Gestational hypertension	80 (1.24)	65 (1.62)	0.94 (0.65,1.34)	0.729	85 (1.34)	1.02 (0.73,1.43)	0.907
Preeclampsia	199 (3.08)	115 (2.87)	0.94 (0.78, 1.19)	0.618	206 (3.24)	0.96 (0.78, 1.19)	0.730
Preterm birth^c^	313 (4.84)	196 (4.90)	**0.76 (0.62, 0.92)**	**0.004**	368 (5.79)	**1.66 (1.41, 1.95)**	**< 0.001**
Primary caesarean section^c^^,^^d^	904 (29.71)	532 (28.39)	0.92 (0.83, 1.01)	0.079	1,000 (31.47)	0.99 (0.91, 1.08)	0.824
Large for gestational age^b^	619 (9.58)	376 (9.40)	1.05 (0.91, 1.21)	0.508	716 (11.3)	0.95 (0.83, 1.07)	0.372
Small for gestational age^b^	408 (6.31)	293 (7.32)	1.17 (1.00, 1.38)	0.051	350 (5.50)	0.95 (0.81, 1.11)	0.488
Low birth weight	250 (3.87)	177 (4.42)	1.29 (0.98, 1.69)	0.070	241 (3.79)	0.77 (0.59, 1.00)	0.053
Macromisca	186 (2.88)	119 (2.97)	1.07 (0.84, 1.37)	0.578	221 (3.48)	0.95 (0.78, 1.20)	0.742
Neonatal intensive care unit admission^c^	1,587 (24.55)	931 (23.26)	0.93 (0.84.1.02)	0.138	1,672 (26.30)	1.07 (0.98, 1.16)	0.149

aWG-F, adequate weight gain in the first trimester; iWG-F, inadequate weight gain in the first trimester; eWG-F, excessive weight gain in the first trimester.

a Adjusted for pre-pregnancy body mass index, maternal age, delivery weeks, parity, and gestational weight gain; preterm birth adjusted for the same confounders except for delivery weeks, and the reference is the adequate weight gain in the first trimester.

b Chi-square test for incidence among three groups, *p* < 0.001.

c Chi-square test for incidence of preterm birth, primary caesarean section, and neonatal intensive care unit admission are 0.032, 0.007, and 0.002, respectively.

d Primary caesarean section ratio is calculated by dividing the number of cesarean deliveries by the number of women without a prior caesarean history. In the three groups, the number of women without a prior cesarean history is 1,874, 3,043, and 3,178.

The meaning of the bold values is that after adjusting for PPBMI, maternal age, delivery weeks (excluding preterm birth), parity, and GWG, iWG-F was linked to a lower incidence of GDM (aOR = 0.76, 95% CI 0.69, 0.84), and preterm birth (aOR = 0.76, 95% CI 0.62, 0.92). In contrast, eWG-F was associated with a high incidence of GDM (aOR = 1.37, 95% CI 1.26–1.49) and preterm birth (aOR = 1.66, 95% CI 1.41–1.95).

### Univariate analysis of clinical factors associated with GDM risk

Factors, significantly associated with an elevated risk of GDM, included PPBMI (OR = 1.06, 95% CI 1.05, 1.07), maternal age (OR = 1.07, 95% CI 1.06, 1.08), parity ≥1 (OR = 1.22, 95% CI 1.15, 1.28), fasting glucose (OR = 1.50, 95% CI 1.38, 1.63) and triglycerides levels (OR = 1.20, 95% CI 1.13, 1.28) in early pregnancy, type 2 diabetes in first-degree relative (OR = 1.52, 95% CI 1.24, 1.86), a history of GDM (OR = 5.53, 95% CI 3.24, 9.79), and WG-F (OR = 1.04, 95% CI 1.03, 1.05). Conversely, WG-S (OR = 0.96, 95% CI 0.95, 0.98), HDL levels (OR = 0.85, 95% CI 0.76, 0.95), and having 12 or more years of education (OR = 0.83, 95% CI 0.73, 0.94) were linked to a reduced risk of GDM. WG before OGTT had no significant association with GDM (*p* = 0.910) (Table 3). The cutoff values were 30.5 years for maternal age, 1.4 mmol/L for triglycerides, 4.9 mmol/L for fasting glucose, and 1.63 mmol/L for HDL.

**Table 3:** Univariate logistic regression analysis of factors related to gestational diabetes.

Clinical factors	OR (95% CI)	*p*
Pregnancy body mass index (kg/m^2^)^a^	1.06 (1.05, 1.07)	<0.001
<18.5	0.79 (0.71, 0.88)	<0.001
18.5–23.9	reference	
24.0–27.9	1.30 (1.18, 1.42)	<0.001
≥28.0	1.57 (1.31, 1.88)	<0.001
Fasting glucose (mmol/L)	1.50 (1.38, 1.63)	<0.001
Triglycerides (mmol/L)	1.20 (1.13, 1.28)	<0.001
Low-density lipoprotein (mmol/L)	1.00 (0.12, 8.13)	1.000
High-density lipoprotein (mmol/L)	0.85 (0.76, 0.95)	0.004
Maternal age (years)	1.07 (1.06, 1.08)	<0.001
Type 2 diabetes in first-degree relative	1.52 (1.24, 1.86)	<0.001
Gestational diabetes history	5.53 (3.24, 9.79)	<0.001
Education level (years)^b^		
<9	reference	
9–12	0.94 (0.82, 1.12)	0.598
≥12	0.83 (0.73, 0.94)	0.004
Parity^c^		
<1	reference	
≥1	1.22 (1.15, 1.28)	< 0.001
Weight gain in the first trimester (kg)	1.04 (1.03, 1.05)	<0.001
Weight gain in the second trimester (kg)	0.96 (0.95, 0.98)	<0.001
Weight gain before the oral glucose tolerance test (kg)	1.00 (0.99, 1.01)	0.910

a The reference is PPBMI 18.5-23.9 kg/m^2^.

b The reference is education level <9 years.

c The reference is parity <1.

### Association of trimester-specific weight gain with GDM incidence across subgroups

Among all participants, using adequate weight gain as the reference, iWG-F was associated with a 14% decrease in GDM incidence (aOR = 0.86, 95% CI 0.78, 0.94) after adjusting for clinical factors significantly associated with GDM identified in the univariate analysis. In contrast, the eWG-F group showed an 18% increase in GDM incidence (aOR = 1.18, 95% CI 1.08, 1.29). Neither WG before OGTT nor WG-S showed a significant association with GDM (*p* > 0.05).

**Figure 2:**
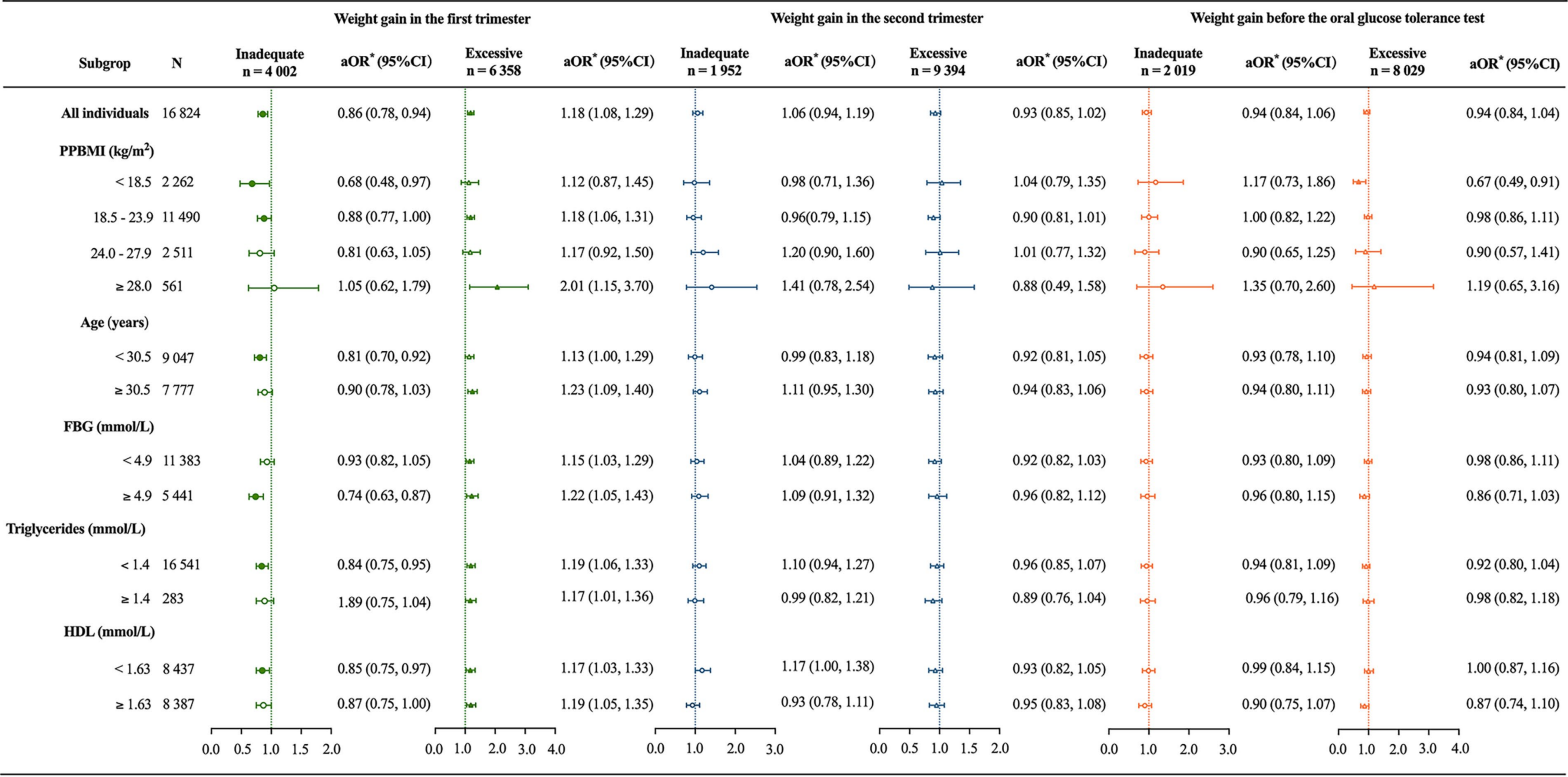
Association of trimester-specific weight gain with gestational diabetes incidence across subgroups. PPBMI, prepregnancy body mass index; FBG, fasting blood glucose; HDL, high-density lipoprotein. *The primary independent variable was categorized weight gain by trimester, and the outcome variable was gestational diabetes mellitus. For all individuals, confounding variables included maternal age, PPBMI, parity, educational level, gestational age at delivery, FBG, triglycerides, and HDL. Subgroup analyses excluded clinical factors specific to each subgroup from the list of confounding variables. Adequate weight gain served as the reference category.

In the subgroup analysis, iWG-F was associated with a 32%, 12%, 19%, 26%, 16%, and 15% reduction in GDM incidence among participants with a PPBMI <18.5 kg/m^2^ (aOR = 0.68, 95% CI 0.48, 0.97), 18.5–23.9 kg/m^2^ (aOR = 0.88, 95% CI 0.77, 1.00), age <30.5 years (aOR = 0.81, 95% CI 0.70, 0.92), fasting glucose ≥4.9 mmol/L (aOR = 0.74, 95% CI 0.63, 0.87), triglyceride <1.4 mmol/L (aOR = 0.84, 95% CI 0.75, 0.95), and HDL <1.63 mmol/L (aOR = 0.85, 95% CI 0.75, 0.97), respectively (Figure 2). eWG-F was associated with an 18%, 101%, and 23% increase in GDM incidence for participants with a PPBMI of 18.5–23.9 kg/m^2^ (aOR = 1.18, 95% CI 1.06, 1.31), ≥28.0 kg/m^2^ (aOR = 2.01, 95% CI 1.15, 3.70), and those aged ≥30.5 years (aOR = 1.23, 95% CI 1.09, 1.40), respectively. Regardless of fasting glucose, triglyceride, or HDL levels in early pregnancy, eWG-F consistently increased the risk of GDM (all *p* < 0.05) (Figure 2). WG-S was not significantly associated with GDM incidence in any subgroup. However, among participants with a PPBMI <18.5 kg/m^2^, excessive WG before OGTT was linked to a decreased incidence of GDM (aOR = 0.67, 95% CI 0.49, 0.91), and this association was not observed in other subgroups (Figure 2).

### Interaction and mediation analyses in the intermediate model

After adjusting for PPBMI, parity, fasting glucose, triglycerides, HDL, age, family history of diabetes, history of GDM, and education level, WG-F (*β* = 0.0418, *p* < 0.001) and WG-S (*β* = −0.0159, *p* = 0.02585) were both significantly associated with the occurrence of GDM. The interaction effect between WG-F and WG-S (*β* = 0.000595, *p* = 0.69680) was insignificant.

**Figure 3:**
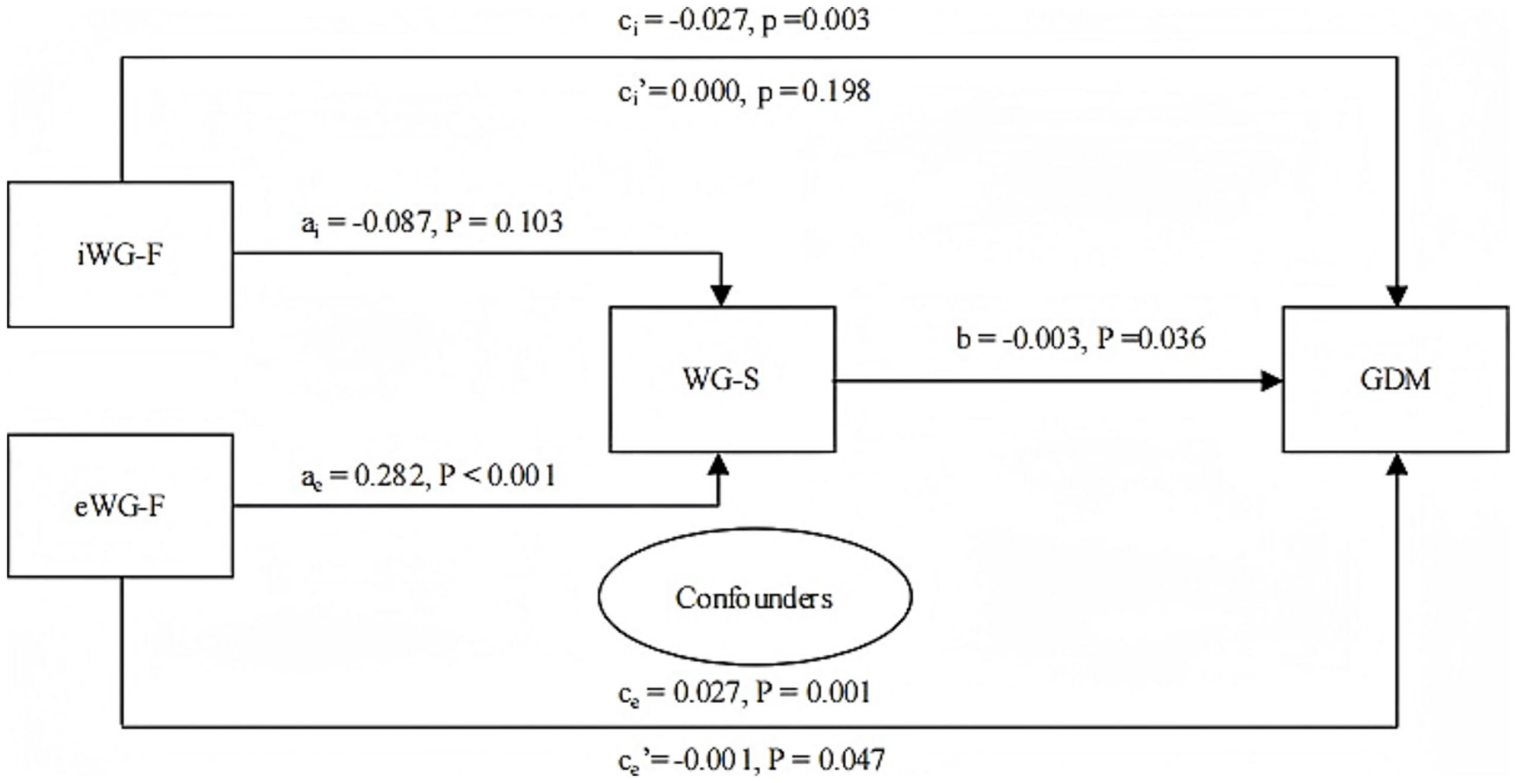
Mediation effect of WG-S on the relationship between iWG-F, eWG-F, and GDM (*n* = 16,824). iWG-F, inadequate weight gain in the first trimester; eWG-F, excessive weight gain in the first trimester; WG-S, weight gain in the second trimester. GDM, gestational diabetes mellitus. Confounders: Prepregnancy body mass index, parity, fasting glucose, triglycerides, high-density lipoprotein, age, family history of diabetes, history of gestational diabetes, and education level. ai: Regression coefficient of iWG-F vs. WG-S; ae: Regression coefficient of eWG-F vs. WG-S; b: Regression coefficient of WG-S vs. GDM; ci: Total effect, regression coefficient when iWG-F vs. GDM (no mediator variable WG-S in the model); ci’: Indirect effect, regression coefficient when iWG-F vs. GDM (mediator variable WG-S in the model); ce: Total effect, regression coefficient when eWG-F vs. GDM (no mediator variable WG-S in the model); ce’: Indirect effect, regression coefficient when eWG-F vs. GDM (mediator variable WG-S in the model).

A mediation effect analysis revealed that the regression coefficient for the total effect for iWG-F was −0.027 (95% CI: −0.044, −0.009; *p* = 0.003). However, the indirect effect was insignificant (*p* = 0.198). For eWG-F, the mediation effect analysis reported regression coefficient of the total effect of 0.027 (95% CI: 0.012, 0.042; *p* = 0.001). The regression coefficient of the indirect effect was −0.001 (95% CI: −0.002), also significant (*p* = 0.000), indicating a partial mediating role of WG-S in the effect of eWG-F on GDM, with an effect proportion of −3.7% (Figure 3).

## Discussion

This study demonstrated a significant association of the first-trimester WG with the incidence of GDM, while no such association was found for the second-trimester and pre-OGTT WG. Inadequate first-trimester weight gain reduces GDM risk, especially in younger women, women with normal or low PPBMI, elevated fasting glucose, and low levels of HDL or triglycerides. This effect does not correlate with abnormal neonatal birth weight.

Excessive WG in the first trimester was shown to increase GDM risk in women with different pre-pregnancy BMI (20–22). However, as reported by Carreno et al. (23) this effect was only observed in women of normal weight. According to Morisset et al. (24) only WG during the first trimester was identified as an independent predictor of GDM, with no association observed for second-trimester weight gain, similarly to other reports (25). Brunner et al. (26) showed that excessive WG before a GDM screening test was linked to higher risk of GDM, while Gibson et al. (27) identified pre-diagnostic WG as a GDM risk factor in overweight or obese women, while no such impact was noted in women of normal BMI or underweight.

In this study, mediation analysis after adjusting for confounders identified eWG-F as a significant predictor of an increased risk of GDM (*β* = 0.027, *p* = 0.001). Subgroup analysis further showed a significance of this association in pregnant women with normal weight or obesity [aOR = 1.18 (95% CI 0.61, 1.31) and 2.01 (95% CI 1.15, 3.70), respectively], but not among underweight or overweight women. Excessive WG before OGTT did not lead to increased GDM risk, no matter maternal age, PPBMI, blood glucose, TG, or HDL levels, which confirms the results of Herring et al. (28).

Among women with lower PPBMI, excessive WG before OGTT correlated with lower GDM risk. Although WG-S was not directly associated with GDM incidence in multivariable logistic regression, there still was a significant negative correlation between WG-S and GDM incidence (*β* = −0.003, *p* = 0.036) in mediation analysis. Additionally, WG-S partly mediated the link between eWG-F and GDM, accounting for −3.7% of the total effect. Despite the minimal effect size, these findings suggest that although WG-S may not independently influence GDM risk, it can modify the impact of excessive early pregnancy WG on the incidence of GDM and potential differences in glucose metabolism across PPBMI categories and pregnancy stages, as well as the complex relationship between WG and GDM risk. Future studies should investigate the mechanisms of this effect.

Early pregnancy is accompanied by an increase in insulin secretory response that precedes changes in insulin sensitivity (12). Insulin resistance initially decreases before rising in mid-to-late pregnancy (29) to support fetal growth. Women who develop late GDM exhibit insufficient insulin secretion and resistance adaptations, potentially impairing hunger and satiety signals (30, 31), which may increase appetite and lead to excessive WG. Excessive WG in early pregnancy predominantly involves adipose tissue (32, 33), which may impair insulin sensitivity, exacerbate hyperglycemia, and disrupt lipid and protein metabolism (34–36). These findings suggest that excessive early pregnancy WG may be a high-risk factor for late-onset GDM.

Changes in lifestyle, such as dietary adjustment and physical exercise, were shown to reduce the risk of GDM by approximately 20% (37–39). Thangaratinam et al. (40) reviewed 44 randomized controlled trials (7,278 women) and demonstrated that lifestyle interventions significantly reduced gestational WG. This aligns with findings by Hill et al. (41) and Mudd et al. (42). In this study, while health education on balanced diets, weight management, and exercise was provided from early pregnancy, 23.79%, 38.42%, and 37.79% of participants experienced inadequate, adequate, and excessive WG-F, with GDM incidences of 24.53%, 26.6%, and 29.5%, respectively (*p* < 0.0001, Table 2). Mediation analysis indicated that iWG-F decreased the risk of GDM regardless of WG-S, consistent with other studies (25, 43, 44). Elevated fasting glucose and low HDL levels in early pregnancy are considered high-risk factors for GDM (45, 46). Subgroup analysis in this study revealed that iWG-F was strongly associated with a reduced incidence of GDM in women with elevated fasting glucose and low HDL, as well as in lower-risk women with normal or underweight PPBMI, younger women, and women with low triglyceride levels. For these low-risk women, weight loss may potentially lead to a higher loss of fat mass, decreasing regional adipose tissue depots, enhancing insulin sensitivity, alleviating insulin resistance (47–49), and potentially lowering the risk of late gestational diabetes. Nandita et al. (50) suggested that inadequate weight gain throughout pregnancy increases the incidence of SGA and LBW. Logistic regression demonstrated a significant association of WG with preterm birth but not with abnormal neonatal weight or NICU admission. These findings emphasize the importance of stage-specific weight management during pregnancy. Future research should explore the mechanisms underlying these associations to refine guidelines for optimizing maternal and neonatal outcomes.

### Strengths and limitations

This study benefits from a large cohort, focusing on weight gain patterns in populations with varying risk during different stages of pregnancy. The findings offer insights for targeted interventions to prevent GDM and may apply to similar maternal health contexts. By combining logistic regression with mediation analysis, the study identified potential roles of the first and second trimesters WG in the development of GDM. Partial mediation of the relationship between second-trimester weight gain and subsequent outcomes provides a basis for future studies to explore underlying pathways.

However, this study has several limitations that should be taken into consideration. First, it only collected short-term outcomes, thus preventing conclusions about the association between GWG and long-term maternal and offspring complications. Second, as an observational study, it cannot establish causal relationships between inadequate WG in the first trimester and maternal or neonatal complications; randomized controlled trials would be needed to determine causality. Third, the data were collected from a single hospital and involved only Chinese women, which limits the generalizability of the results. Additional studies involving diverse populations and multi-centre data are recommended. Fourth, pre-pregnancy weight was measured at home and self-reported, which may have introduced inaccuracies and biases in the data.

## Conclusion

First-trimester WG is significantly linked to the incidence of GDM, while second-trimester and pre-OGTT weight gain show minimal or no significant association. Inadequate first-trimester weight gain reduces the risk of GDM, particularly in subgroups characterized by younger age, normal or underweight PPBMI, elevated fasting glucose, low HDL, or low triglyceride levels, without increasing abnormal neonatal birth weight. Early pregnancy may represent a critical window for GDM prevention. Encouraging minimal or no WG during the early gestation period may be a feasible and acceptable strategy for managing GDM risk.

## Data Availability

The data generated and analyzed during the current study are not publicly available due to privacy protection and ethical restrictions. Requests to access these datasets should be directed to LZ, 18060117656@163.com.
